# Management of pediatric ‘cannot intubate, cannot oxygenate’

**DOI:** 10.1002/ams2.305

**Published:** 2017-08-18

**Authors:** Yohei Okada, Wataru Ishii, Norio Sato, Hirokazu Kotani, Ryoji Iiduka

**Affiliations:** ^1^ Department of Emergency and Critical Care Medicine Japanese Red Cross Society Kyoto Daini Hospital Kyoto Japan; ^2^ Department of Primary Care and Emergency Medicine Kyoto University Hospital Sakyo‐ku Kyoto Japan; ^3^ Department of Forensic Medicine Kyoto University Graduate School of Medicine Sakyo‐ku Kyoto Japan

**Keywords:** Angioedema, cannot ventilate, cricothyroidotomy, difficult airway, surgical airway

## Abstract

**Case:**

“Cannot intubate, cannot oxygenate” (CICO) is a rare, life‐threatening situation. We describe a pediatric case of CICO and highlight some educational points.A 3‐year‐old boy who collapsed in the bathtub came to our emergency department. On admission, he went into cardiac arrest probably because of an airway obstruction. We judged his condition as CICO and carried out an emergent tracheostomy after several attempts to perform a cricothyroidotomy failed. We continued resuscitation; however, circulation did not return spontaneously.

**Outcome:**

The child died, and the autopsy showed an airway obstruction caused by idiopathic anaphylaxis or acquired angioedema.

**Conclusion:**

This case highlights that it can be anatomically difficult to perform a percutaneous cannula cricothyroidotomy and scalpel cricothyroidotomy safely in pediatric CICO cases. An emergent tracheostomy using the scalpel–finger–bougie technique on the proximal trachea should be considered in such cases.

## Introduction

“Cannot intubate, cannot oxygenate” (CICO) is a rare but life‐threatening situation.^1^ In pediatric CICO, the Difficult Airway Society (DAS) guideline suggests some airway rescue techniques.^2^, ^3^ However, pediatric CICO is very rare, and this suggestion is mostly based on animal experimental results and expert opinions;^3^ therefore, further reports are necessary to consider the best strategy for pediatric CICO. We describe a pediatric case of CICO and highlight some educational points.

## Case

**Figure 1:**
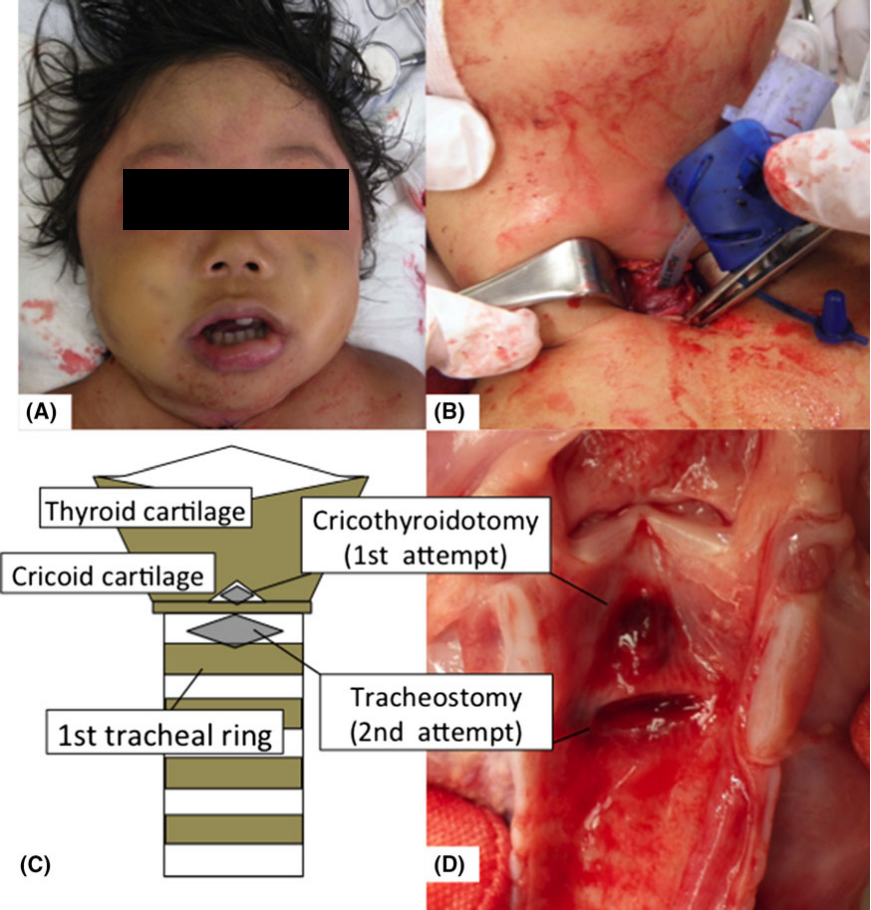
Pediatric case of “cannot intubate, cannot oxygenate”. A, Extreme swelling of the lip, tongue, and lower jaw. B, Tracheostomy below the cricoid cartilage with a vertical incision added to the transverse incision. C, D, Schema and anatomical specimens of the larynx and trachea in this case.

**Figure 2:**
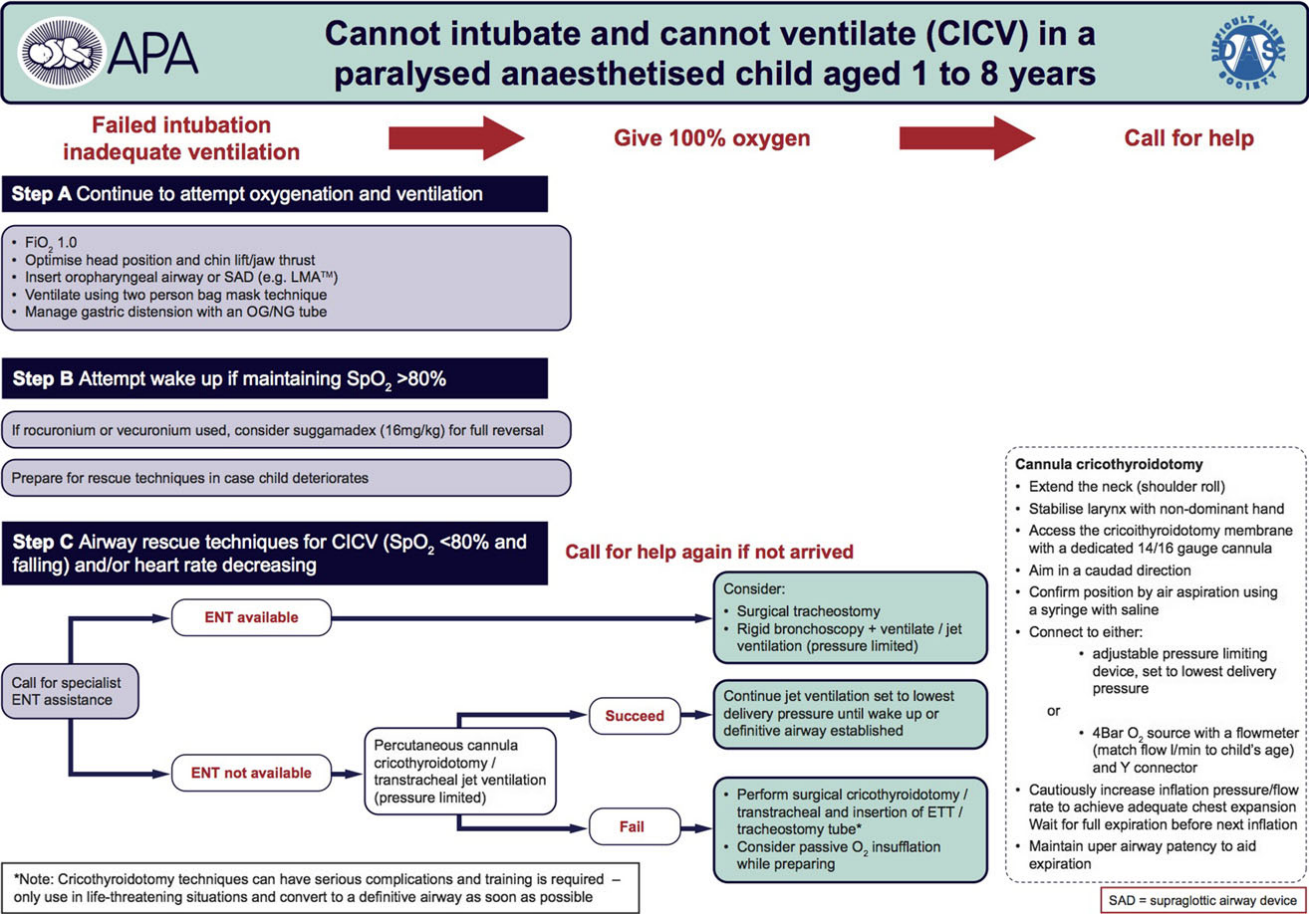
Guidelines for the management of “cannot intubate, cannot oxygenate” when there is failure to intubate and adequately ventilate an anesthetized and paralyzed child aged 1–8 years.

A 3‐year‐old boy who was previously healthy with no known allergies collapsed in a bathtub. When emergency medical service arrived to him 5 min after the collapse, he was in cardiac arrest (pulseless electrical activity). They immediately started basic life support and attempted to secure the airway by chin lift or jaw thrust but they could not obtain it due to the intraoral edema. He came to our emergency department approximately 20 min after the collapse. On admission, he was still in cardiac arrest (pulseless electrical activity) with obvious swelling of the lip, tongue, and lower jaw (Fig. 1). Bag–valve–mask ventilation was attempted, but intraoral edema completely disturbed his ventilation. We immediately attempted intubation of his trachea by using a conventional laryngoscope and a video laryngoscope but failed. Consequently, we judged his situation to be CICO. At first, we considered percutaneous cannula cricothyroidotomy (PCC) with angiocatheter needle, but we were afraid of penetrating the posterior tracheal wall because we could not find enough working space or a proper angle to safely perform percutaneous needle insertion (Fig. 2). Nonetheless, we started to perform cricothyroidotomy (SC) 2 min after the admission.

His neck was extended by pushing a pillow under his shoulders. Identifying the cricothyroid membrane and stabilizing the larynx with the left hand, we made a transverse skin incision through the skin and cricothyroid membrane. We used a cricothyroidotomy kit (Mini‐Trach II – Non Seldinger Kit; Smiths Medical, Minneapolis, Minnesota, USA), and the introducer in the kit was inserted through the incision in the cricothyroid membrane. Subsequently, we tried to insert the kit cannula into the trachea, but it would not insert because the space between the cricothyroid cartilages was smaller than the outer diameter of the cannula. Several attempts were made with the cannula for 2 min after we started SC (Fig. 1). However, the cannula was kinked in the space between the cricothyroid cartilages, and we could not provide ventilation. Subsequently, we removed the cannula.

An approximate 2‐cm vertical midline skin incision was made and followed by blunt dissection to identify the trachea. A transverse incision below the cricoid cartilage was made as a tracheostomy. After the introducer was inserted through the incision, the cannula was inserted into the trachea (Fig. 1). We finally reached an open airway and provided ventilation 10 min after we began the surgical attempt to secure the airway. We gave 0.01‐mg/kg adrenaline by intraosseous route 11 times based on the pediatric advanced life support, but the swelling did not resolve. We did not use other medications, such as steroids or local vasoconstrictions, to reduce the edema.

We continued resuscitation, but spontaneous circulation did not return and the patient died. The autopsy showed that the airway obstruction was caused by idiopathic anaphylaxis or acquired angioedema^4^ and that the diameter of the space between the thyroid cartilage and cricoid cartilage was approximately 3 mm.

## Discussion

**Figure 3:**
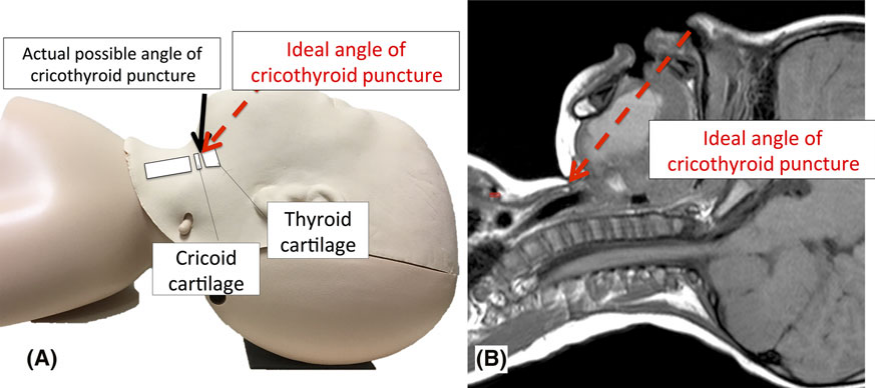
Treatment of pediatric “cannot intubate, cannot oxygenate” cases. A, Model of the approach used in percutaneous cannula cricothyroidotomy. Neck extension in small children does not provide a sufficient angle to safely perform percutaneous cricothyroidotomy. B, Magnetic resonance image of a 3‐year‐old boy whose case of “cannot intubate, cannot oxygenate” is reported of here. This image shows insufficient working space or angle to safely perform percutaneous cricothyroidotomy.

This pediatric CICO case highlights some educational points that should be considered in pediatric CICO. The DAS guideline suggests that if an ear–nose–throat (ENT) doctor is not available, PCC is recommended (Fig. 2).^3^ Percutaneous cannula cricothyroidotomy and a jet ventilation system with a long expiratory time are well established in adult emergency airway management.^5^ It could buy some time until ENT or extracorporeal membrane oxygenation services are available. However, we believe that PCC is technically difficult in pediatric CICO cases and has some risk to cause severe complications. First, it is anatomically too difficult to obtain a sufficient angle of approach to carry out PCC safely in small children. Neonates and infants generally have short fat necks. Thus, when performing a PCC, the steeper the angle of approach to the cricothyroid membrane, the greater the likelihood of a posterior tracheal puncture, which is a severe complication.^2^ In our case, full extension of the head and neck still did not allow a sufficient flattening to undertake PCC safely during the resuscitation (Fig. 3). Furthermore, even in adults, PCC with jet ventilation can be associated with significant complications, including surgical emphysema, pneumothorax, and lung injury, and it is believed that the pediatric population is particularly at risk of these complications.^3^ Moreover, some experts suggest that PCC should not be used in children aged <6 years and surgical cricothyroidotomy/tracheostomy should be preferred because the trachea is far smaller than in adults and risking misplacement and posterior tracheal wall injury.^3^ Because of these factors, we think that PCC is not appropriate in pediatric CICO.

The DAS guideline suggests that SC should be selected if PCC fails.^3^ However, we think that SC is also not feasible in pediatric patients because there are some special anatomical features to be considered in children. For example, the cricothyroid membrane in adults is large enough (average, 13.7 mm long and 12.4 mm wide) to identify and to allow an incision to be made easily, but in children, it is much smaller. Especially in neonates, it has a mean length of only 2.6 ± 0.7 mm and a width of 3.0 ± 0.63 mm.^6^ Such a small cricothyroid membrane limits the size of a device that may be safely passed with minimal damage to the larynx.^7^ The outer diameter of the cannula we used was 5.4 mm, whereas the width of the space was approximately 3 mm, which explains why we could not pass the cannula through the space smoothly and why the cannula kinked while attempting insertion. Moreover, identification of landmarks and performing a cricothyroidotomy are more technically difficult in neonates or small infants than in adults^6^ because the hyoid bone and cricoid cartilages are often more prominent than the thyroid cartilage, and the thyroid cartilage rides underneath the hyoid bone.

Above all, before PCC and SC were attempted, we should have considered if an emergent tracheostomy was feasible. In published reports, tracheostomy by an ENT doctor was successful in three cases of CICO,^3^ and the guideline for pediatrics suggests that an emergent tracheostomy should be considered as the first step if an ENT doctor is available. However, it is uncommon for an ENT doctor to always be available in such emergent situations. In such cases, an acute care surgeon with a lot of experience performing tracheostomies should decide if an emergent tracheostomy is feasible.

We suggest some modifications when an emergent tracheostomy is attempted in pediatric patients. The incision of the tracheostomy should be made below the cricoid cartilage more proximal than is common in tracheostomies in adults. Because the distal anatomy may be more difficult to define than the proximal anatomy, the more distal attempts to obtain a clear airway along the trachea in pediatric patients may lower the success rates for both cannula and scalpel techniques.^2^ Moreover, we should select the scalpel–finger–bougie technique, which is recommended in the guideline for cricothyroidotomy in adults if the cricothyroid membrane is impalpable or if other techniques have failed.^1^ An emergent tracheostomy can also be carried out in pediatric patients^2^ as follows: a midline vertical incision is made with a scalpel from thyroid cartilage to the upper edge of sternum, soft tissue is dissected bluntly by finger. A transverse stab incision of the trachea is made, the bougie introducer is inserted into the trachea, and the tube is inserted into the trachea. This incision is more invasive, but it enables the surgeon to identify the anatomy more quickly and clearly and stabilize the larynx and tracheae more constantly than standard tracheostomy by an ENT doctor. Furthermore, using a bougie is also useful to insert the tube smoothly into the pediatric small trachea in emergency situations. Thus, we think that this procedure is more valuable than standard tracheostomy in pediatric CICO, particularly for patients aged <6 years, because pediatric trachea is far smaller, more mobile, flaccid, and easily compressible than in adults.^3^

In our case, a vertical incision was added to the transverse incision and bluntly dissected, which enabled us to define the anatomy below the cricoid cartilage and insert the introducer and tube into the trachea as a tracheostomy (Fig. 1). Unfortunately, it took 10 min to obtain the airway; however, if we had knowledge of pediatric emergent tracheostomy and had attempted the modified procedure first, a clear airway would have been obtained in a few minutes. Therefore, we suggest that this technique enables identification of the anatomy and completion of pediatric emergent tracheostomies immediately and safely.

## Conclusion

This case highlights that PCC and SC are difficult to undertake safely in pediatric CICO cases and that emergent tracheostomy at the proximal trachea using the scalpel–finger–bougie technique should be considered.

## Disclosure

Informed consent was obtained from the subject's parent for publication of this case report.

Conflict of interest: Authors declare no Conflict of Interests for this article.
